# The Association between Breastfeeding Duration and Lipid Profile among Children and Adolescents

**DOI:** 10.3390/nu13082728

**Published:** 2021-08-08

**Authors:** Yanhui Li, Di Gao, Li Chen, Tao Ma, Ying Ma, Manman Chen, Bin Dong, Yanhui Dong, Jun Ma, Luke Arnold

**Affiliations:** 1 Institute of Child and Adolescent Health, School of Public Health, Peking University, Beijing 100191, China; yanhui_lyh@163.com (Y.L.); gaodi1993@163.com (D.G.); clcl@bjmu.edu.cn (L.C.); 1610306216@pku.edu.cn (T.M.); mypku232@163.com (Y.M.); 1911210173@pku.edu.cn (M.C.); majunt@bjmu.edu.cn (J.M.); 2 Department of Data Governance and Analytics, South Western Sydney Primary Health Network, Campbelltown, NSW 2560, Australia; luke.arnold@swsphn.com.au

**Keywords:** breastfeeding, breast milk, lipid, dyslipidemia, children and adolescents

## Abstract

To investigate the relationship between breastfeeding duration and lipid profile among children and adolescents, a cross-sectional survey using random cluster sampling was performed, and a national sample of 12,110 Chinese children and adolescents aged 5–19 years were collected. Breastfeeding duration and sociodemographic factors were collected by questionnaires. Fasting blood samples were obtained to test the lipid profile. Linear regression and logistic regression models were employed to evaluate the association between breastfeeding duration and lipid profile. We found that prolonged breastfeeding was related with a low level of total cholesterol (TC), LDL-C, HDL-C, and TC/HDL-C in children and adolescents. With an increased duration of breastfeeding, the magnitude of the association between breastfeeding and lipid profile enlarged. The levels of TC, LDL-C, HDL-C, and TC/HDL-C in participants who were breastfed for more than 12 months decreased by 6.225 (95% CI: −8.390, −4.059), 1.956 (95% CI: −3.709, −0.204), 1.273 (95% CI: −2.106, −0.440) mg/dL, and 0.072 (95%CI: −0.129, −0.015), respectively, compared with those who were not breastfed. The corresponding risk of high TC declined by 43% (aOR: 0.570, 95% CI: 0.403, 0.808). The association was similar in both boys and girls, but only statistically significant in children and young adolescents aged 5–14 years. This suggested that prolonged breastfeeding duration was related with low lipid levels and decreased abnormal lipid risk, especially in children and young adolescents. These findings support the intervention of prompting a prolonged duration of breastfeeding to improve the childhood lipid profile.

## 1. Introduction

Dyslipidemia is an important risk factor for coronary artery disease and stroke, which plays a vital role in the pathophysiological mechanisms of cardiovascular disease [1]. Dyslipidemia, once considered in adults, now has a high prevalence in children and adolescents, with approximately one in five having dyslipidemia [2,3]. It can be progressive throughout childhood into adulthood, and young people who have abnormal lipid levels may be at risk of cardiovascular disease in later life. In addition, dyslipidemia also contributes to endothelial dysfunction in children, an early stage of atherosclerotic lesion. As atherosclerotic changes during childhood are potentially reversible [4], early identification and intervention of dyslipidemia in children and adolescents may improve longer-term health outcomes. Data on early life determinants of dyslipidemia suggest that breast milk is a protective factor [5].

Breast milk provides the perfect nutrition and biologically active components for infants. Breastfeeding has clear short-term benefits for child health, which prevents many perinatal complications associated with preterm labor and reduces morbidity and mortality [6]. In recent years, accumulated studies have focused on the long-term health outcomes of breastfeeding. Breastfeeding can reduce the rates of hypertension, insulin resistance, and metabolic syndrome in adolescence, and prevent the development of cardiometabolic diseases in adults [6]. Evidence from observational studies showed that those who were breastfed had a lower level of total cholesterol in later life [7]. Breast milk has higher total cholesterol content in contrast to formula (90–150 mg/L VS. 0–4 mg/L) [8], and its effect may be related to reducing the endogenous cholesterol synthesis rate (3-fold lower in breast milk) [9], however, the association between breastfeeding and lipid profile was not consistent. A systematic review found that mean total cholesterol in childhood and adolescence was not related to infant feeding patterns [10]. There was also no clear association between breastfeeding duration and serum lipid levels at the age of 18 years [11]. However, a randomized controlled trial reported that infants who were breastfeeding had more favorable plasma lipid profile at 13–16 years [12]. A recent birth cohort study also indicated that exclusive breastfeeding at 0 to 3 months was associated with a healthier lipid profile in late adolescence [13]. The reason for inconsistent results may be that these studies did not take both breastfeeding duration and the age of final examination into consideration. Some studies revealed that the composition and concentrations of lipid in breast milk alter over a single feed, as well as over lactation [14]. Therefore, breastfeeding duration may have different magnitudes of health effect, and exploring the relationship between breastfeeding duration and lipid profile in children and adolescents of different ages could aid the intervention strategy aimed to reduce the risk of dyslipidemia and improve cardiovascular health.

In this study, we investigated the relationship between breastfeeding duration and lipid profile among children and adolescents aged 5–19 years using data from a large cross-sectional study conducted in 7 provinces in China, with the aim to provide evidence on the health effect of prolonging breastfeeding duration in children and adolescents.

## 2. Materials and Methods

### 2.1. Study Population

Baseline data of a national multi-centered cluster randomized controlled trial among Chinese children and adolescents in 2013 were used. Participants came from 7 provinces, including Tianjin, Liaoning, Shanghai, Hunan, Guangdong, Chongqing, and Ningxia. Between 12–16 primary and secondary schools were enrolled in each province, and 2 classes were randomly selected from every grade in each school. Details about this study and the sampling method have been published previously [15]. Among 16,750 participants aged 5–19 years whose blood samples were available, 4640 participants with missing data of breastfeeding were excluded, and the final sample size for this study was 12,110. The study has been approved by the ethical committee of the Peking University (number: IRB0000105213034), and written informed consent was obtained from both students and their parents.

### 2.2. Assessment of Sociodemographic Variables and Duration of Breastfeeding

Sociodemographic variables, including age, sex, region (urban or rural), dietary behavior (consumption of sugar-sweetened beverage and meats, and frequency of high-energy food and fried food intake), and physical activity were collected by a children questionnaire. Dietary consumption was derived from students’ self-reported frequency (days) and amount (serving) of the food they had during the past 7 days. Students also reported their vigorous-intensity and moderate-intensity activity frequency (days) and duration (minutes) during the past 7 days, to calculate the average daily vigorous-intensity and moderate-intensity activity time, and daily physical activity time is the sum of the two. The parental questionnaire was additionally performed to collect the information on parental education and feeding type. Parents were also asked to provide information on feeding type (breastfeeding or not), as well as the duration of breastfeeding (in month), and the participants were divided into four breastfeeding duration groups: non-breastfeeding, 0–6 months, 6–12 months, and >12 months.

### 2.3. Detection of Indicators and Outcome Variables

Height and weight were measured using a portable stadiometer (model TZG, China) and lever type weight scale (model RGT-140, China), with the accuracy of 0.1 cm and 0.1 kg, respectively. Body mass index (BMI) was calculated as weight (kg) divided by squared height (m^2^).

Blood samples were obtained by venipuncture after fasting for 12 h. Samples were centrifuged at 3000 rpm for 10 min, serum was collected and stored at −80 °C. All biochemical analyses on blood were carried out at a biomedical analyses company, which is accredited by Peking University [15]. The lipid levels, including total cholesterol (TC), triglyceride (TG), low-density lipoprotein cholesterol (LDL-C), and high-density lipoprotein cholesterol (HDL-C), were measured by an autoanalyzer (TBA-120FR, Toshiba, Tokyo, Japan). TC and TG were assayed by enzymatic method, while LDL-C and HDL-C were measured using clearance method. TC/HDL-C was calculated as TC (mg/dL) divided by HDL-C (mg/dL).

Abnormal lipid was defined as follows [16]: High TC referred to TC ≥ 200 mg/dL. High TG was considered as TG ≥ 100 mg/dL in children 9 years old or younger, and ≥130 mg/dL in adolescents 10 years old and older. While high LDL-C was defined as LDL-C ≥ 130 mg/dL, low HDL-C was regarded as HDL-C < 40 mg/dL. A participant with one or more abnormal lipid levels was defined as having dyslipidemia.

### 2.4. Statistical Analysis

All analyses were performed by SPSS 24.0 (IBM Corporation, New York, NY, USA). Analysis of variance was used for continuous variables and chi-squared test was applied for categorical data, to detect the differences among different breastfeeding duration groups. Bonferroni methods were used for multiple comparison. Linear regression model was applied to assess the relationship between breastfeeding duration and lipid levels, and logistic regression model was employed to estimate the association of breastfeeding duration and the risk of abnormal lipid. Confounding factors, including region, sex, age, parental education level, and child’s information (such as BMI, physical activity, sugar-sweetened beverage consumption, meat consumption, and frequency of high-energy food and fried food intake), were adjusted in the adjusted model. *p* value for trend was also reported to evaluate time-response correlation. All statistical tests were two sided and were considered statistically significant at *p* < 0.05.

## 3. Results

A total of 6061 girls and 6049 boys were included in this study, and the prevalence of dyslipidemia was 27.4%. The characteristics of participants are shown in Table 1 by breastfeeding groups. Differences in age, BMI, region, parental educational level, and lipid profile between breastfeeding groups were detected. Children and adolescents who had prolonged breastfeeding duration were associated with lower levels of TC, LDL-C, HDL-C, and TC/HDL-C; a lower prevalence of high TC; and a higher prevalence of low HDL-C.

**Figure 1:**
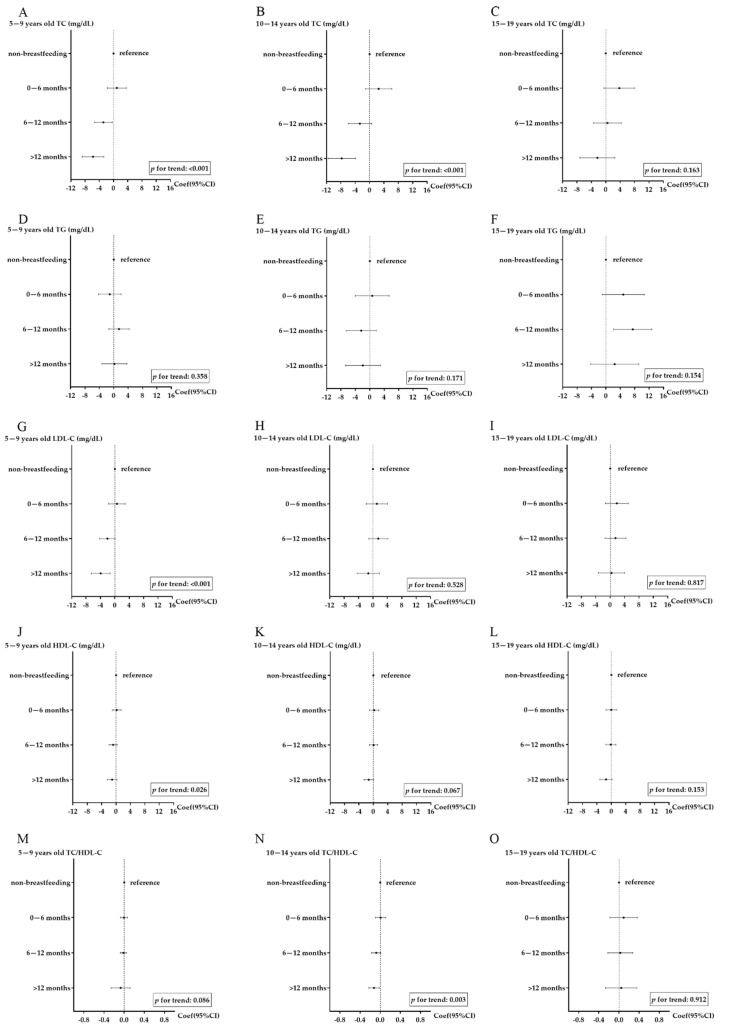
The differences in lipid levels among different breastfeeding groups, stratified by age group. (**A**): association between breastfeeding duration and total cholesterol (TC) in children aged 5–9 years old; (**B**): association between breastfeeding duration and TC in children aged 10–14 years old; (**C**): association between breastfeeding duration and TC in children aged 15–19 years old; (**D**): association between breastfeeding duration and triglyceride (TG) in children aged 5–9 years old; (**E**): association between breastfeeding duration and TG in children aged 10–14 years old; (**F**): association between breastfeeding duration and TG in children aged 15–19 years old; (**G**): association between breastfeeding duration and low-density lipoprotein cholesterol (LDL-C) in children aged 5–9 years old; (**H**): association between breastfeeding duration and LDL-C in children aged 10–14 years old; (**I**): association between breastfeeding duration and LDL-C in children aged 15–19 years old; (**J**): association between breastfeeding duration and high-density lipoprotein cholesterol (HDL-C) in children aged 5–9 years old; (**K**): association between breastfeeding duration and HDL-C in children aged 10–14 years old; (**L**): association between breastfeeding duration and HDL-C in children aged 15–19 years old; (**M**): association between breastfeeding duration and TC/HDL-C in children aged 5–9 years old; (**N**): association between breastfeeding duration and TC/HDL-C in children aged 10–14 years old; (**O**): association between breastfeeding duration and TC/HDL-C in children aged 15–19 years old. Notes: adjusted for region, sex, parental education level, children’s BMI, physical activity, sugar-sweetened beverage consumption, meat consumption, and frequency of high-energy food and fried food.

Table 2 shows the differences in lipid levels among various breastfeeding duration groups before and after adjustment. In the crude model (model 1), longer breastfeeding duration was associated with enlarged differences in levels of TC, LDL-C, HDL-C, and TC/HDL-C when the non-breastfeeding group was considered as the reference (*p* for trend < 0.001). After adjusting for confounders, the differences in lipid levels between non-breastfeeding and other breastfeeding groups were still significant, though the differences were narrowed. Breastfeeding for more than 12 months was related with 6.225 (95% CI: −8.390, −4.059), 1.956 (95% CI: −3.709, −0.204), 1.273 (95% CI: −2.106, −0.440) mg/dL lower levels of TC, LDL-C, and HDL-C, respectively, and 0.072 (95% CI: −0.129, −0.015) lower levels of TC/HDL-C compared with the non-breastfeeding group after full adjustment. In stratified analysis, similar relationships between breastfeeding duration and lipid levels were observed in boys and girls (Supplementary Figure S1). However, the differences were mainly found in age groups of 5–14 years (*p* < 0.05), rather than in those aged 15–19 years (Figure 1).

**Figure 2:**
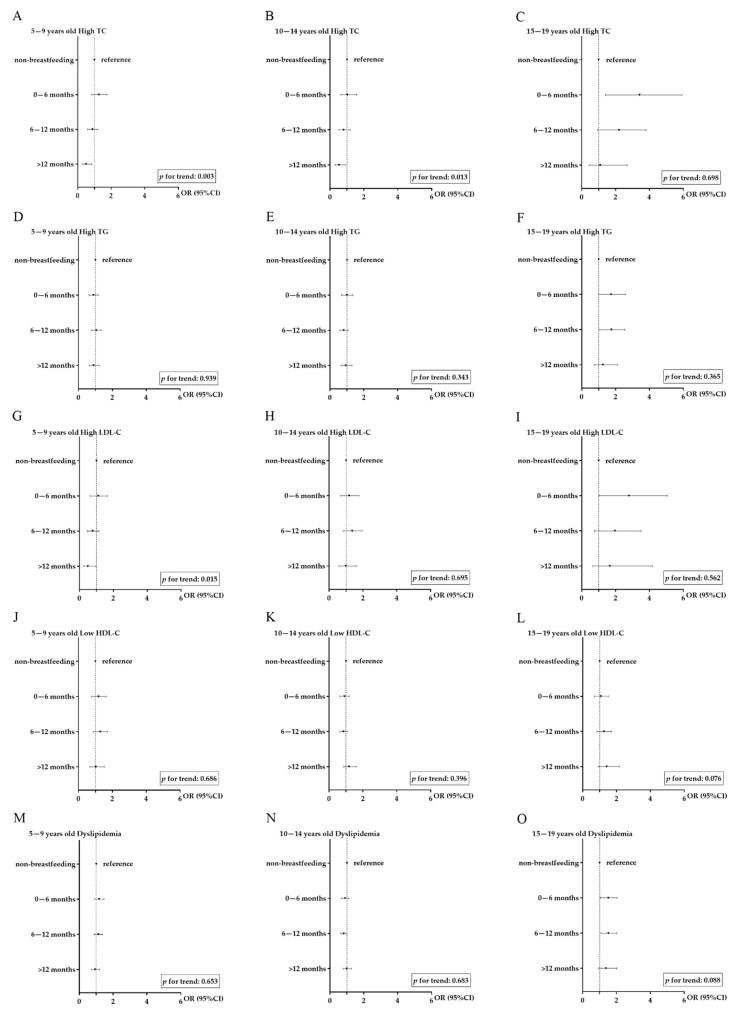
The risk of abnormal lipid among different breastfeeding groups, stratified by age group. (**A**): association between breastfeeding duration and high total cholesterol (TC) in children aged 5–9 years old; (**B**): association between breastfeeding duration and high TC in children aged 10–14 years old; (**C**): association between breastfeeding duration and high TC in children aged 15–19 years old; (**D**): association between breastfeeding duration and high triglyceride (TG) in children aged 5–9 years old; (**E**): association between breastfeeding duration and high TG in children aged 10–14 years old; (**F**): association between breastfeeding duration and high TG in children aged 15–19 years old; (**G**): association between breastfeeding duration and high low-density lipoprotein cholesterol (LDL-C) in children aged 5–9 years old; (**H**): association between breastfeeding duration and high LDL-C in children aged 10–14 years old; (**I**): association between breastfeeding duration and high LDL-C in children aged 15–19 years old; (**J**): association between breastfeeding duration and low high-density lipoprotein cholesterol (HDL-C) in children aged 5–9 years old; (**K**): association between breastfeeding duration and low HDL-C in children aged 10–14 years old; (**L**): association between breastfeeding duration and low HDL-C in children aged 15–19 years old; (**M**): association between breastfeeding duration and dyslipidemia in children aged 5–9 years old; (**N**): association between breastfeeding duration and dyslipidemia in children aged 10–14 years old; (**O**): association between breastfeeding duration and dyslipidemia in children aged 15–19 years old. Notes: adjusted for region, sex, parental education level, children’s BMI, physical activity, sugar-sweetened beverage consumption, meat consumption, and frequency of high-energy food and fried food.

Consistent with the results of lipid levels, after adjusting for confounders, the risk of high TC decreased with the increase in breastfeeding duration (*p* for trend < 0.001). The risk of high TC was reduced by 43% (aOR: 0.570, 95% CI: 0.403, 0.808) in the group of breastfeeding more than 12 months, compared with the non-breastfeeding group (Table 3). There were no significant differences in high LDL-C and low HDL-C among various breastfeeding duration groups. In stratified analysis, a prolonged breastfeeding duration was associated with a lower risk of high TC, mainly in girls and participants aged 5–14 years (Supplementary Figure S2 and Figure 2).

## 4. Discussion

In this large population-based study with 12,110 participants aged 5 to 19 years, we found that breastfeeding was related to a better lipid profile, and children and adolescents with a history of prolonged breastfeeding had more desirable lipid profiles compared with those who were non-breastfed. A similar pattern of association was observed in both sexes, but mainly in children and young adolescents.

Accumulated studies have reported the association between breastfeeding and lipid profile in children and adolescents, but their results were inconsistent. In line with the present study, cross-sectional and longitudinal studies conducted in Iran, UK, US, Mexico, and Hong Kong showed that breastfeeding had a beneficial effect on lipid profile among children and adolescents [5,12,13,17,18]. Conversely, a systematic review showed that there was no difference in cholesterol between breast and bottle-feeding in children and adolescents aged 1–16 years [10]. A population-based birth cohort study also failed to observe significant differences in lipid levels at 12 years between breastfed and never breastfed children [19]. Inconsistencies across studies may reflect residual confounding, sample size, age range, and other factors [20,21].

In the present study, we found that prolonged breastfeeding duration was related to low lipid levels and a decreased risk of abnormal lipid. Consistent with our research reporting the protective effects of prolonged breastfeeding, a recent study showed that breastfeeding duration was inversely associated with hypertriglyceridemia, low HDL-C, and metabolic syndrome in adolescents aged 10–15 years [22]. The national population-based study was conducted among Iranian students ages 10–18 years, and also showed that participants who were breastfed the longest had lower serum levels of TC, LDL, and TG, than those who were not breastfed. Although the differences did not reach statistical significance in LDL and TG, there was a marginally significantly correlation between breastfeeding duration with the means of TC level [23]. On the contrary, in a cluster randomized controlled trial, an intervention to promote increased duration of exclusive breastfeeding did not influence cardiometabolic risk factors at age 11.5 years [24]. The differences in existing findings may be related to socioeconomic background, specific study design, and adjustment of potential confounding factors.

The benefit of long breastfeeding duration mainly focused on TC. The association between breastfeeding duration and low HDL-C was no longer statistically significant after adjustment, which indicated that the elevated risk of low HDL-C may be related to participants’ BMI and lifestyle. Studies have found that participants who successfully lost weight during the lifestyle intervention were associated with better HDL-C, not TC, TG, or LDL-C [25]. Lifestyle may have an effect on HDL-C, by changing the cholesterol efflux capacities and having different HDL subspecies [26,27]. These results suggested that lifestyle and weight may have a greater impact on HDL-C than breastfeeding.

The biological mechanisms related to the beneficial effects of breastfeeding remain unclear. A plausible explanation may be related to specific or complex components and molecular distribution in human milk. Breast milk is rich in bioactive factors, which have been found to reduce oxidative stress, promote organ maturation, enhance immunity, and promote gastrointestinal function [6,28]. The dominant phospholipid and fat globule were different in breast milk and formula, which may affect the lipid digestion and absorption [28,29]. The distribution of fatty acids along glycerol influences their availability, with palmitic acid at the sn-2 position being absorbed more readily [30]. A large proportion of palmitic acid is esterified to the sn-2 position of the triglyceride in breast milk [31]. This position has been observed to influence the infants’ plasma lipid profile, including cholesterol concentration [32]. In addition, studies found that cholesterol synthesis rate was inversely correlated with dietary cholesterol intake [9]. This could involve changes in the expression of 3-hydroxy-3methylglutaryl coenzyme A (HMG-CoA) reductase, the rate regulating enzyme for endogenous cholesterol synthesis [9]. A high level of cholesterol in breast milk is most likely to promote subsequent healthy lipid profile by downregulating the HMG-CoA reductase and reducing the endogenous cholesterol synthesis rate. What’s more, the composition and concentration of breast milk alter dramatically over lactation [32]. Some studies have shown that the concentration of fat and energy in breast milk has a positive correlation with lactation from 1st to the 48th month [33]. Thus, long breastfeeding duration may have a stronger inhibiting effect on endogenous cholesterol synthesis. Previous studies have shown a dose-response relationship between the consumption of human milk and ratios of LDL-C to HDL-C, and apoB to apoA-1 [12]. The levels of bioactive factors and antioxidant capacity of breast milk also seem to decrease along the lactation period [6]. Therefore, the longer breastfeeding duration could lead to a healthier lipid profile.

Furthermore, our findings suggested that the protective effect of breastfeeding on the childhood lipid profile attenuated with increasing age. A prospective study found that exclusive and predominant breastfeeding were associated with low TC levels in children of 4 years old [17]. Randomized controlled studies also revealed that participants who had been randomized to a banked breastmilk group had lower levels of LDL/HDL ratio, TC, and LDL-C compared with those who received preterm formula at 13–16 years, although these differences were marginally significant [12]. Similarly, there also has been evidence to show that the effect of breastfeeding could not affect the lipid profile in late adolescents and adults [11,34]. However, some studies have reported the protective effect of breastfeeding on older people [13,35]. More study is warranted to clarify whether the influence of breastfeeding on health attenuates with age.

There are some limitations to our study. First, the cross-sectional analysis cannot determine causality. Second, some potential confounders, such as mothers’ dietary behavior during breastfeeding and children’s eating pattern after weaning and during childhood, were not included, since maternal dietary may affect the composition and concentration of breast milk lipid, feeding patterns may influence the children’s food preference, and breastfeeding mothers usually were more prone to provide healthy food to their children. Third, we did not further identify the role of breastfeeding duration longer than 12 months. Future studies covering longer periods of breastfeeding duration are needed. Fourth, we did not distinguish exclusive breastfeeding and mixed breastfeeding; it is therefore possible that the relationship between breastfeeding duration and lipid profile is being underestimated.

## 5. Conclusions

This study found a time-response relationship between breastfeeding duration and lipid profile in children and adolescents, and prolonged breastfeeding duration was associated with a healthy lipid profile, especially for the risk of high TC. Breastfeeding is a modifiable factor, hence, these findings support the intervention strategy of prolonged breastfeeding (more than 12 months) aimed to reduce the risk of dyslipidemia in children and adolescents. In addition, the findings also suggest that the protective effect of breastfeeding is related to age, and the beneficial effect was mainly shown in children and young adolescents. In practice, more attention and health education should be paid to older adolescents, to promote lipid metabolism and cardiovascular health.

## Figures and Tables

**Table 1:** Characteristics of participants by different breastfeeding groups.

Characteristics	Breastfeeding Groups				*p*
	Non-Breastfeeding (*n* = 1997)	0–6 Months (*n* = 2783)	6–12 Months (*n* = 5153)	>12 Months (*n* = 2177)	
Age, year	10.7 ± 3.3	10.7 ± 3.3	11.0 ± 3.2 ^#^	11.0 ± 3.1 ^#^	0.001
Height, cm	146.0 ± 17.4	145.7 ± 17.2	146.5 ± 17.0	147.3 ± 15.7 ^#^	0.008
Weight, kg	41.1 ± 16.2	40.4 ± 15.8	41.3 ± 15.3	42.8 ± 15.9 ^#^	<0.001
BMI, kg/m^2^	18.6 ± 3.9	18.3 ± 3.7 ^#^	18.6 ± 3.7	19.1 ± 4.1 ^#^	<0.001
Physical activity ≥ 1h/d	515 (29.9%)	744 (30.5%)	1428 (31.6%)	591 (32.4%)	0.314
Urban area	1323 (66.2%)	1649 (59.3%) ^#^	2925 (56.8%) ^#^	920 (42.3%) ^#^	<0.001
Girls	957 (47.9%)	1377 (49.5%)	2643 (51.3%)	1084 (49.8%)	0.065
Daily SSB consumption ≤ 1 serving ^$^	1648 (89.8%)	2357 (90.2%)	4357 (90.8%)	1796 (90.3%)	0.592
Daily meat consumption, serving^$^					<0.001
<2	1475 (78.8%)	1930 (73.3%)	3859 (78.7%)	1777 (87.2%)	
2–3	302 (16.1%)	573 (21.8%)	837 (17.1%)	189 (9.3%)	
>3	96 (5.1%)	130 (4.9%)	209 (4.3%)	73 (3.6%)	
Frequency of high-energy food intake					0.220
0 time	506 (26.7%)	679 (25.6%)	1212 (24.5%)	556 (27.0%)	
1–3 times per week	1063 (56.0%)	1511 (57.0%)	2801 (56.7%)	1138 (55.2%)	
≥4 times per week	329 (17.3%)	462 (17.4%)	931 (18.8%)	367 (17.8%)	
Frequency of fried food intake					<0.001
0 time	807 (42.7%)	1251 (47.3%)	2164 (43.8%)	817 (39.9%)	
1–3 times per week	957 (50.7%)	1214 (45.9%)	2424 (49.1%)	1052 (51.3%)	
≥4 times per week	125 (6.6%)	182 (6.9%)	350 (7.1%)	180 (8.8%)	
Maternal education level					<0.001
Primary or below	146 (7.5%)	159 (5.8%)	508 (10.1%)	282 (13.2%)	
Secondary or equivalent	1526 (78.8%)	2046 (74.9%)	3977 (79.0%)	1709 (80.0%)	
Bachelor degree or above	265 (13.7%)	527 (19.3%)	552 (11.0%)	146 (6.8%)	
Paternal education level					<0.001
Primary or below	111 (5.7%)	130 (4.8%)	344 (6.8%)	237 (11.1%)	
Secondary or equivalent	1509 (77.5%)	1993 (73.1%)	4031 (80.0%)	1755 (82.0%)	
Bachelor degree or above	326 (16.8%)	603 (22.1%)	666 (13.2%)	149 (7.0%)	
TC, mg/dL	155.1 ± 33.2	156.7 ± 32.7	151.4 ± 33.2 ^#^	146.6 ± 30.2 ^#^	<0.001
TG, mg/dL	80.8 ± 40.4	79.1 ± 42.4	82.1 ± 39.9	83.3 ± 44.7	0.002
LDL-C, mg/dL	85.2 ± 27.0	85.3 ± 26.9	84.1 ± 26.4	80.5 ± 25.0 ^#^	<0.001
HDL-C, mg/dL	53.5 ± 12.9	53.9 ± 13.2	53.1 ± 12.4	51.3 ± 12.2 ^#^	<0.001
TC/HDL-C	3.0 ± 0.9	3.0 ± 0.8	3.0 ± 0.9 ^#^	3.0 ± 0.8 ^#^	0.002
High TC	139 (7.0%)	215 (7.7%)	278 (5.4%)	69 (3.2%) ^#^	<0.001
High TG	266 (13.3%)	326 (11.7%)	673 (13.1%)	310 (14.2%)	0.065
High LDL-C	116 (5.8%)	157 (5.6%)	277 (5.4%)	92 (4.2%)	0.081
Low HDL-C	255 (12.8%)	344 (12.4%)	673 (13.1%)	376 (17.3%) ^#^	<0.001
Dyslipidemia	545 (27.3%)	746 (26.8%)	1391 (27.0%)	636 (29.2%)	0.209

BMI: body mass index, SSB: sugar-sweetened beverage, TC: total cholesterol, TG: triglyceride, LDL-C: low-density lipoprotein cholesterol, HDL-C: high-density lipoprotein cholesterol, TC/HDL-C: total/HDL cholesterol ratio. Continuous variables were expressed by mean values and standard deviation, and categorical variables were expressed by numbers and percentages. ^$^ One serving of SSBs is approximately 250 mL. One serving of meat is approximately 50 g. ^#^ indicated there was a statistically significant difference compared with the non-breastfeeding group.

**Table 2:** Multiple linear regression analysis of breastfeeding duration and lipid levels, β (95% CI).

Models	Serum Lipid	Breastfeeding Duration	*p*	Breastfeeding Group			
				Non-Breastfeeding	0–6 Months	6–12 Months	>12 Months
Model 1	TC (mg/dL)	−0.555 (−0.652, −0.457)	<0.001	0 (reference)	1.687 (−0.184, 3.558)	−3.675 (−5.357, −1.993) **	−8.505 (−10.482, −6.528) **
	TG (mg/dL)	0.228 (0.104, 0.352)	<0.001	0 (reference)	−1.677 (−4.061, 0.706)	1.327 (−0.816, 3.469)	2.508 (−0.010, 5.026)
	LDL-C (mg/dL)	−0.276 (−0.355, −0.197)	<0.001	0 (reference)	0.164 (−1.353, 1.680)	−1.071 (−2.433, 0.292)	−4.693 (−6.295, −3.091) **
	HDL-C (mg/dL)	−0.132 (−0.170, −0.094)	<0.001	0 (reference)	0.485 (−0.243, 1.212)	−0.388 (−1.042, 0.265)	−2.156 (−2.924, −1.387) **
	TC/HDL-C	−0.004 (−0.007, −0.002)	0.002	0 (reference)	−0.006 (−0.056, 0.043)	−0.067 (−0.112, −0.023) **	−0.060 (−0.112, −0.008) *
Model 2	TC (mg/dL)	−0.466 (−0.564, −0.368)	<0.001	0 (reference)	1.024 (−0.817, 2.865)	−3.104 (−4.760, −1.447) **	−7.477 (−9.441, −5.513) **
	TG (mg/dL)	0.150 (0.024, 0.276)	0.019	0 (reference)	−1.583 (−3.955, 0.789)	0.361 (−1.774, 2.496)	1.574 (−0.956, 4.105)
	LDL-C (mg/dL)	−0.143 (−0.223, −0.064)	<0.001	0 (reference)	0.879 (−0.623, 2.380)	−0.127 (−1.478, 1.225)	−2.282 (−3.884, −0.680) **
	HDL-C (mg/dL)	−0.106 (−0.145, −0.067)	<0.001	0 (reference)	0.510 (−0.218, 1.239)	−0.096 (−0.751, 0.560)	−1.768 (−2.545, −0.991) **
	TC/HDL-C	−0.004 (−0.007, −0.001)	0.004	0 (reference)	−0.021 (−0.071, 0.029)	−0.072 (−0.117, −0.027) **	−0.062 (−0.115, −0.009) *
Model 3	TC (mg/dL)	−0.394 (−0.501, −0.286)	<0.001	0 (reference)	1.876 (−0.112, 3.864)	−1.957 (−3.754, −0.161) *	−6.201 (−8.353, −4.048) **
	TG (mg/dL)	0.184 (0.043, 0.325)	0.011	0 (reference)	−0.376 (−2.986, 2.233)	1.323 (−1.035, 3.681)	2.478 (−0.347, 5.304)
	LDL-C (mg/dL)	−0.109 (−0.196, −0.021)	0.015	0 (reference)	0.959 (−0.658, 2.576)	0.112 (−1.349, 1.573)	−1.821 (−3.572, −0.071) *
	HDL-C (mg/dL)	−0.097 (−0.140, −0.055)	<0.001	0 (reference)	0.499 (−0.285, 1.283)	−0.099 (−0.808, 0.610)	−1.686 (−2.536, −0.837) **
	TC/HDL-C	−0.003 (−0.006, 0.000)	0.055	0 (reference)	−0.005 (−0.059, 0.049)	−0.044 (−0.093, 0.005)	−0.044 (−0.103, 0.015)
Model 4	TC (mg/dL)	−0.399 (−0.507, −0.290)	<0.001	0 (reference)	2.175 (0.170, 4.180) *	−1.864 (−3.674, −0.055) *	−6.225 (−8.390, −4.059) **
	TG (mg/dL)	0.064 (−0.073, 0.201)	0.360	0 (reference)	0.849 (−1.685, 3.383)	1.744 (−0.543, 4.031)	0.666 (−2.070, 3.403)
	LDL-C (mg/dL)	−0.128 (−0.216, −0.041)	0.004	0 (reference)	1.329 (−0.294, 2.953)	0.261 (−1.204, 1.726)	−1.956 (−3.709, −0.204) *
	HDL-C (mg/dL)	−0.069 (−0.111, −0.028)	0.001	0 (reference)	0.174 (−0.598, 0.945)	−0.201 (−0.897, 0.495)	−1.273 (−2.106, −0.440) **
	TC/HDL-C	−0.005 (−0.008, −0.002)	0.001	0 (reference)	0.021 (−0.032, 0.074)	−0.034 (−0.081, 0.014)	−0.072 (−0.129, −0.015) *

Model 1 did not adjust for any factors. Model 2 adjusted for region, sex, age, and parental education level. Model 3 further adjusted for physical activity, sugar-sweetened beverage consumption, meat consumption, and frequency of high-energy food and fried food. Model 4 further adjusted for BMI. * *p* < 0.05, ** *p* < 0.01.

**Table 3:** Logistic regression analysis of breastfeeding duration and abnormal lipid risk, OR (95% CI).

Models	Abnormal Lipid	Breastfeeding Duration	*p*	Breastfeeding Group			
				Non-Breastfeeding	0–6 Months	6–12 Months	>12 Months
Model 1	High TC	0.954 (0.941, 0.967)	<0.001	1 (reference)	1.119 (0.897, 1.397)	0.762 (0.618, 0.941) *	0.438 (0.326, 0.588) **
	High TG	1.007 (0.999, 1.016)	0.101	1 (reference)	0.863 (0.726, 1.027)	0.978 (0.839, 1.139)	1.081 (0.906, 1.289)
	High LDL-C	0.982 (0.969, 0.996)	0.012	1 (reference)	0.969 (0.757, 1.241)	0.921 (0.737, 1.152)	0.716 (0.540, 0.947) *
	Low HDL-C	1.021 (1.013, 1.030)	<0.001	1 (reference)	0.964 (0.810, 1.146)	1.026 (0.879, 1.198)	1.426 (1.201, 1.694) **
	Dyslipidemia	1.005 (0.999, 1.012)	0.114	1 (reference)	0.976 (0.857, 1.110)	0.985 (0.877, 1.106)	1.100 (0.961, 1.259)
Model 2	High TC	0.963 (0.949, 0.976)	<0.001	1 (reference)	1.105 (0.882, 1.384)	0.811 (0.655, 1.005)	0.501 (0.371, 0.678) **
	High TG	1.005 (0.996, 1.014)	0.281	1 (reference)	0.877 (0.735, 1.047)	0.966 (0.826, 1.128)	1.048 (0.873, 1.259)
	High LDL-C	0.993 (0.978, 1.008)	0.344	1 (reference)	1.071 (0.829, 1.385)	1.005 (0.796, 1.268)	0.881 (0.655, 1.185)
	Low HDL-C	1.018 (1.009, 1.028)	<0.001	1 (reference)	0.967 (0.809, 1.155)	0.992 (0.846, 1.163)	1.366 (1.142, 1.634) **
	Dyslipidemia	1.006 (0.999, 1.013)	0.105	1 (reference)	0.993 (0.870, 1.134)	0.985 (0.874, 1.109)	1.114 (0.969, 1.281)
Model 3	High TC	0.969 (0.953, 0.984)	<0.001	1 (reference)	1.276 (0.990, 1.644)	0.935 (0.734, 1.191)	0.561 (0.397, 0.791) **
	High TG	1.007 (0.996, 1.017)	0.214	1 (reference)	0.939 (0.772, 1.141)	0.993 (0.834, 1.182)	1.091 (0.888, 1.340)
	High LDL-C	0.994 (0.977, 1.011)	0.513	1 (reference)	1.165 (0.867, 1.563)	1.080 (0.825, 1.416)	0.899 (0.634, 1.275)
	Low HDL-C	1.016 (1.006, 1.027)	0.002	1 (reference)	0.948 (0.780, 1.152)	0.986 (0.828, 1.174)	1.291 (1.057, 1.578) *
	Dyslipidemia	1.006 (0.998, 1.014)	0.124	1 (reference)	1.045 (0.903, 1.210)	1.014 (0.889, 1.158)	1.126 (0.962, 1.318)
Model 4	High TC	0.968 (0.953,0.984)	<0.001	1 (reference)	1.342 (1.036, 1.739) *	0.945 (0.737, 1.212)	0.570 (0.403, 0.808) **
	High TG	0.999 (0.988, 1.010)	0.837	1 (reference)	0.999 (0.814, 1.226)	1.009 (0.840, 1.211)	0.976 (0.787, 1.212)
	High LDL-C	0.993 (0.976, 1.010)	0.406	1 (reference)	1.252 (0.925, 1.695)	1.126 (0.852, 1.487)	0.885 (0.618, 1.268)
	Low HDL-C	1.011 (1.001, 1.022)	0.033	1 (reference)	0.986 (0.806, 1.207)	0.999 (0.833, 1.197)	1.196 (0.972, 1.472)
	Dyslipidemia	1.002 (0.994, 1.010)	0.658	1 (reference)	1.092 (0.938, 1.271)	1.024 (0.892, 1.175)	1.056 (0.897, 1.243)

Model 1 did not adjust for any factors. Model 2 adjusted for region, sex, age, parental education level. Model 3 further adjusted for physical activity, sugar-sweetened beverage consumption, meat consumption, frequency of high-energy food and fried food. Model 4 further adjusted for BMI. * *p* < 0.05, ** *p* < 0.01.

## Data Availability

The data supporting the conclusions of this article will be made available from the corresponding author upon request.

## Supplementary Materials

The supplementary material published online alongside the manuscript. The following are available online at https://www.mdpi.com/article/10.3390/nu13082728/s1, Supplementary Figure S1: the differences of lipid levels among different breastfeeding groups, stratified by sex, Supplementary Figure S2: the risk of abnormal lipid among different breastfeeding groups, stratified by sex.
