# Supplementary figures and images for: The Association between Breastfeeding Duration and Lipid Profile among Children and Adolescents
Source: Nutrients. 2021 Aug 8;13(8):2728. doi: 10.3390/nu13082728 (PMC8401711; doi:10.3390/nu13082728)

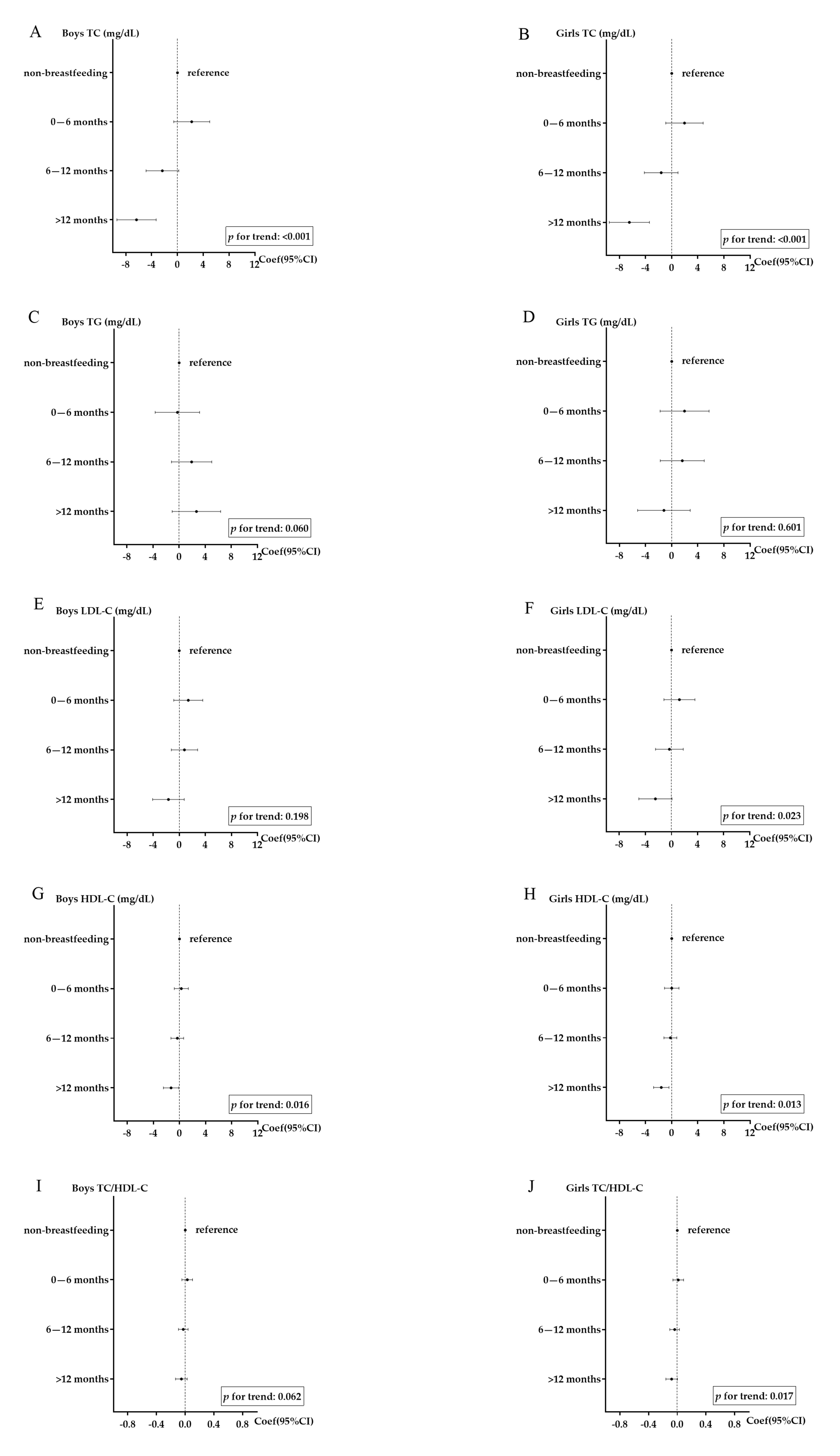

Supplement: Supplementary file 1 [file nutrients-13-02728-s001.zip › supplementary files/Supplementary Figure S1-new.tif]

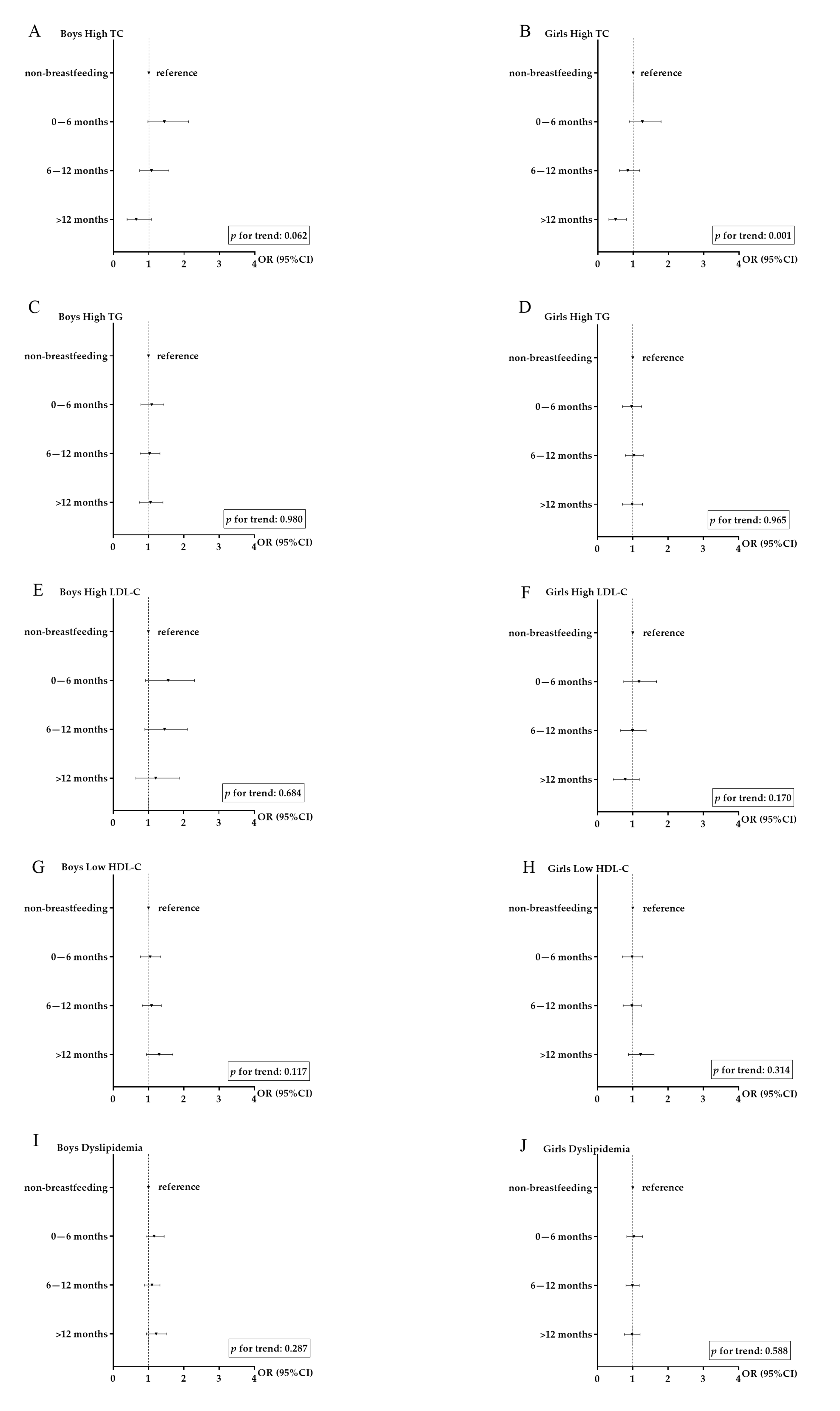

Supplement: Supplementary file 1 [file nutrients-13-02728-s001.zip › supplementary files/Supplementary Figure S2-new.tif]
